# Chemosensory Communication of Gender Information: Masculinity Bias in Body Odor Perception and Femininity Bias Introduced by Chemosignals During Social Perception

**DOI:** 10.3389/fpsyg.2015.01980

**Published:** 2016-01-20

**Authors:** Smiljana Mutic, Eileen M. Moellers, Martin Wiesmann, Jessica Freiherr

**Affiliations:** Diagnostic and Interventional Neuroradiology, Uniklinik RWTH AachenAachen, Germany

**Keywords:** sex, sexual dimorphism, sex recognition, mood, body odor, olfaction

## Abstract

Human body odor is a source of important social information. In this study, we explore whether the sex of an individual can be established based on smelling axillary odor and whether exposure to male and female odors biases chemosensory and social perception. In a double-blind, pseudo-randomized application, 31 healthy normosmic heterosexual male and female raters were exposed to male and female chemosignals (odor samples of 27 heterosexual donors collected during a cardio workout) and a no odor sample. Recipients rated chemosensory samples on a masculinity-femininity scale and provided intensity, familiarity and pleasantness ratings. Additionally, the modulation of social perception (gender-neutral faces and personality attributes) and affective introspection (mood) by male and female chemosignals was assessed. Male and female axillary odors were rated as rather masculine, regardless of the sex of the donor. As opposed to the masculinity bias in the odor perception, a femininity bias modulating social perception appeared. A facilitated femininity detection in gender-neutral faces and personality attributes in male and female chemosignals appeared. No chemosensory effect on mood of the rater was observed. The results are discussed with regards to the use of male and female chemosignals in affective and social communication.

## Introduction

Humans, although seen as the most highly scented apes (Stoddart, 1990), have been less extensively studied compared to non-primate mammals when it comes to chemosensory communication. Nonetheless, human chemosignalling research has revealed that stable and temporal features of a sender are communicated through the chemical senses (Lübke and Pause, 2015). Especially, when male and female communication via axillary odor is studied, features of a sender affect various levels in a receiver, e.g., social behavior (Frumin et al., 2015), emotional perception (Zhou and Chen, 2009; Albrecht et al., 2011), memory function (Alho et al., 2015), social evaluation (Mitro et al., 2012), attractiveness, and mating preferences (Wedekind and Füri, 1997; Thornhill et al., 2003; Havlicek et al., 2005).

Research on gender-related differences in chemosensory communication reveals the impact of chemosignals on sexual attraction and mate choice (Doty and Cameron, 2009). Chemosensory mate perception is largely affected by female relationship status and menstrual cycle phase (Havlicek et al., 2005; Rantala et al., 2006) as well as intake of hormonal contraception (Roberts et al., 2008). In a study on the chemosensory effect on sexual attraction and mate choice in male raters, it was found that male raters can distinguish between ovulating and non-fertile female body odor (Kuukasjärvi et al., 2004) and that they display higher testosterone levels during exposure to an ovulating female’s body odor (Miller and Maner, 2009). Female odor raters explicitly value male body odor pleasantness (Herz and Cahill, 1997; Herz and Inzlicht, 2002) and are able to assess male attractiveness and fluctuating asymmetry, a marker of developmental stability (Thornhill and Gangestad, 1999).

More importantly, features of the receiver such as biological sex and sexual orientation (Sergeant et al., 2007; Lübke et al., 2012), hormonal status (Roberts et al., 2008) or chemosensory sensitivity to chemosignals (Pause et al., 1999) influence the chemosensory communication process. Nevertheless, body odor sampling studies with both male and female donors are still rare. Mere sex discrimination ability based on female and male axillary odor has been examined in previous research (Russell, 1976; Hold and Schleidt, 1977; Schleidt, 1980; Doty, 1981) stating that humans are able to marginally discriminate between male and female axillary odor. Male body odor is perceived as more musky (Russell, 1976), more intense and less pleasant than female body odor (Hold and Schleidt, 1977; Doty et al., 1978; Schleidt et al., 1981; Mitro et al., 2012). It has further been established that higher chemosensory discrimination of body odors is more frequent for female raters and that the body odor of the opposite sex is expected to smell more pleasant (Hold and Schleidt, 1977; Sergeant, 2010; Mitro et al., 2012).

Ample evidence is pointing to sex-specific differences in male and female body odor. Chemical analyses of volatile compounds in axillary sweat provide information about distinct chromatographic profiles of male and female samples (Penn et al., 2007), and even non-volatile odor precursors of axillary sweat (fatty acids and thiols) were shown to vary concentration-wise in a sex-specific manner (Troccaz et al., 2009). These findings support the idea that sex-related body odor differences do not only exist but can be also communicated among individuals.

Besides natural axillary odor, chemical compounds that are most commonly supposed to have a communicative function are applied to explore chemosensory communication of sex information (e.g., Gustavson et al., 1987; Jacob and McClintock, 2000; Savic et al., 2001; Wysocki and Preti, 2004; Grammer et al., 2005; Lundström et al., 2006; Olsson et al., 2006; Wyart et al., 2007; Zhou et al., 2014) and were shown to affect masculinity and femininity ratings of schematic body movements (Zhou et al., 2014).

Concluding from chemosensory research on sex and gender communication, the conveyed chemosensory information seems to be modulated by the sex of the sender (donor) and the receiver (rater). Taking both factors into account is crucial for an accurate investigation of the still poorly understood chemosensory effect of male and female body odor on higher cognition, emotion, and behavior in a receiver. As it was shown that sex and age of a donor induce rapid mood changes in receivers (e.g., Chen and Haviland-Jones, 1999), affective and social communication via the chemical senses can only be accurately examined in case modulating effects of communicated gender information from the sender to the receiver are known. A study applying the putative chemosignal androstadienone to male and female participants (Hummer and McClintock, 2009) revealed that emotional information processing was altered during its exposure compared to a control odor (clove). While subliminal face processing and perception of emotional words was affected by androstadienone, emotional introspection (mood) was not affected. This finding relates to the discussion by Grammer et al. (2005) of whether chemosignals rather influence socially oriented perception of conspecifics (e.g., evaluation of others, sexual attractant) or self-perception (e.g., as mood enhancer or modulator) in human chemosignalling in general as well as during chemosensory gender communication. This question had not been considered before.

Therefore, we aim to systematically examine the chemosensory information emitted from male and female donors to male and female raters in odor perception, social perception and emotional introspection. We hypothesize that, in a chemosensory rating task, male and female chemosensory samples produce distinguishable intensity and pleasantness ratings. In a masculinity-femininity rating task, we expect male and female chemosensory samples to be correctly assigned by a collective of male and female raters. Furthermore, and beyond mere communication of sex information, we explore whether body odors modulate social or self-perception. As our chemosensory samples convey social information, we expect the perception of social stimuli (in a personality rating task) and conspecifics (in face and word rating tasks) to be modulated rather than introspection (mood rating). More precisely, regarding the rating gender-neutral personality attributes and faces, we expect female chemosensory samples to be associated with a femininity bias and male chemosensory samples to be associated with a masculinity bias.

## Materials and Methods

### Participants

The present study was carried out in accordance with the recommendations of the Ethics committee of the medical faculty of RWTH Aachen University and in accordance with the Declaration of Helsinki with written informed consent provided from all participants. In total, 32 healthy participants (raters) took part in the experiment. Participants were healthy, heterosexual (Martins et al., 2005) non-smokers with no current medication or drug intake (Doty and Bromley, 2004). All eligible participants rated their current sexual behavior (past 12 months) as exclusively heterosexual on the 7-point Kinsey Scale (Kinsey et al., 1948), ranging from 0 (exclusively heterosexual with no homosexual behavior) to 6 (exclusively homosexual with no heterosexual behavior). To ensure that no exogenous odors contaminate the body odors, dietary and hygienic instructions for 2 days prior to the experiment included abstinence from alcohol, caffeine, garlic, onions, spices and the use of deodorants, body fragrances and lotions (Albrecht et al., 2011). Participants showered with scent-free body wash and shampoo, did not shave the armpits and refrained from visiting public pools and saunas. Female participants were scheduled to always participate in the same phase of their menstrual cycle. All females stated to be non-pregnant, did not take hormonal contraception, had experienced a regular menstrual cycle during the six months preceding their participation and were always tested in the same cycle phases each (follicular phase: *n* = 4, periovulatory phase: *n* = 5 and luteal phase: *n* = 7). Phases were defined as a count of post-menstrual onset days based on self-report (Lundström et al., 2006) e.g., for a menstrual cycle length of 28 days, we defined follicular phase from day 1 to 11, periovulatory phase from day 12 to 16 and luteal phase from day 17 to 28. One participant was excluded due to a lack of task compliance. The final sample consisted of *n* = 31 participants (age range = 19–47 years), including 15 males (*M* = 27.80 years, *SD* = 8.83 years) and 16 females (*M* = 29.56 years, *SD* = 9.33 years). The two groups did not differ in age, *t*(29) = 0.539, *p* = 0.594. The odor identification test MONEX-40 (Freiherr et al., 2012) classified all participants as normosmic (*M* = 32.20, *SD* = 2.77; range = 26–38).

### Donation Procedure and Chemosensory Samples

In total, 29 healthy participants (donors) took part in the body odor donation. Female participants did not take hormonal contraception, experienced regular menstrual cycles and stated to be non-pregnant. Donors underwent the same dietary and hygienic instructions as the raters. Two participants were excluded due to acute medication intake prior to the experiment and blood circulation problems. The final sample consisted of *n* = 27 participants (range = 20–49 years), including 14 males (*M* = 25.93 years; *SD* = 8.87 years) and 13 females (*M* = 26.31 years; *SD* = 7.54 years). The two groups did not differ in age, *t*(25) = 0.119, *p* = 0.906.

Upon arrival, participants were informed about the purpose of the study and screened for dietary and hygienic compliance. After cleaning their armpits with scent-free wipes, cotton pads were attached under both armpits. Participants wore a long-sleeved cotton shirt washed with scent-free detergent. In order to increase sweat production, all participants wore a synthetic raincoat. They exercised in a training room with room temperature on an ergometer for 20 min with 100 W/h and 60–80 cycles per minute. After a short break, the pads were replaced and the donation procedure was repeated. Before and after each donation, pulse and blood pressure were assessed (Omron IntelliTM sense, R7 HEM-632-E2; Omron Healthcare Co., Ltd. Kyoto, Japan). To ascertain a correct value, three measurements were performed (immediately after one another) and their mean was used as a final value. This resulted in three measurements in total: before the first 20 min donation, during the break between the first and the second 20 min donation and after the second 20 min donation. Upon successful completion of the donation, the donors were paid 20 Euros.

Immediately after donation, the chemosensory pads underwent an olfactory examination by the experimenter. Pads were not included in case the body odor was not free from perceivable exogenous odors (such as perfume, smoke or spices) or unusual odor intensity was detected (*n* = 2). To minimize odor contamination, pad handling was performed after disinfection of utensils and hands with isopropanol (70%). Each pad was cut in sixteen parts (quadrants of 1 cm × 1 cm). Male and female superdonor pools were created to assure homogeneous odor samples within the experimental groups and to reduce effects of individual variations. This method has been successfully used in prior donation studies (Albrecht et al., 2011; Dalton et al., 2013), which utilized large donor sample groups. Control samples of odorless clean cotton pads (no odor samples without chemosignals) were created and treated like the chemosensory samples in terms of cutting and freezing. The samples were kept in re-sealable storage bags at -80°C (Lenochova et al., 2009) for no longer than 5 months. Thus bacterial decomposition of the samples was avoided.

### Application Procedure

Raters were invited to three experimental application sessions (within-subject design) within 3 months (one session every 28 days). Females were scheduled to always participate in the same phase of their menstrual cycle. In a double-blind randomized design, participants were exposed to one of the three chemosensory samples (male chemosignals, female chemosignals, and neutral odor) per application session. Thirty minutes before application, quadrants of four donors were randomly chosen from the superdonor pool and put in cotton filter masks. At the beginning and at the end of each of the three application sessions, participants’ mood was assessed via self-rating (Watson et al., 1988). The response options were adapted to a 100-point VAS (0 = not at all or very slightly, 25 = a little, 50 = moderately, 75 = quite a bit, 100 = extremely) and mood before and after exposure to the chemosensory samples was compared. After the fitting of the mask under the noses of the participants (Albrecht et al., 2011), a familiarization phase of five minutes was applied to avoid influences of imminent hormonal changes in association with the odor presentation onset that potentially modulate the participants’ task performance (Wyart et al., 2007). The experimental tasks took 20–25 min and the odor mask was removed after exactly 30 min of odor exposure. Participants were instructed to breathe normally and rate the masculinity-femininity dimension of the chemosignals, of personality-attributes and of faces. All tasks were computerized. Upon successful completion of all three testing appointments, the raters were paid 45 Euros.

#### Odor Perception Tasks

A three-alternative forced-choice test was performed at the beginning of the first session to evaluate odor discrimination capacities. Participants indicated blindly among three samples (two distractors vs. one target sample) the one sample smelling differently with three repetitions of all target and distractor combinations (four discriminations per odor condition and twelve discriminations in total).

At the end of the last session, participants performed an odor-rating task where the odor dimensions masculinity-femininity, intensity, and pleasantness were assessed. Hedonic ratings included assessment of intensity, pleasantness, and familiarity of the chemosensory samples (male chemosignals, female chemosignals and no odor sample), and were performed on 100-point VAS ranging from 0 (*not intense/pleasant/familiar at all*) to 100 (*extremely intense/pleasant/familiar*). The masculinity-femininity ratings of the chemosensory samples were performed using 100-point VAS ranging from the endpoint *masculin*e (0), to neutral, (50) to the endpoint *feminine* (100).

#### Social Perception Tasks

For the adjective-rating task, participants rated the masculinity and femininity of 20 gender-neutral adjectives describing persons and personality traits on 100-point VAS.

The gender-neutrality of the personality attributes was identified in a pilot study with 20 male (*n* = 10) and female participants (*n* = 10) evaluating the neutrality of 60 adjectives (Pauly et al., personal communication). This sample included masculinity-related personality attributes (e.g., brutal) as well as femininity-related personality attributes (e.g., caring). The participants rated the gender of the words on a 5-point rating scale with the endpoints very masculine (-2) and very feminine (2). In total, 20 gender-neutrally rated adjectives describing personality attributes (*M* = 0 ± 1 SD) were included in the task (e.g., friendly, childish, discrete).

**FIGURE 1:**
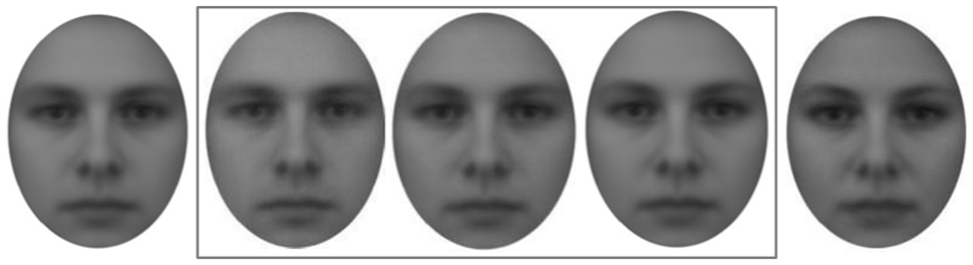
**Stimuli for the gender-neutral face rating task.** The left face depicts a 100% masculine face template. The right face depicts a 100% female face template. Boxed faces in center depict merged gender-neutral experimental stimuli with different proportions (40% male + 60% female, 50% each, 60% male + 40% female). Reprint of facial stimuli with kind permission from Daniel Lundqvist. Taken and modified from Lundqvist and Litton (1998).

For the rating of the faces, gender-neutral faces were constructed using the female and male face of the Averaged Karolinska Directed Emotional Faces repertoire (Lundqvist and Litton, 1998) that are not expressing emotions (picture codes: MNES and FNES). Then, hair in both pictures was masked so that only facial features were visible (**Figure 1**). Subsequently, the male and female facial stimuli were merged with three different proportions (40% male + 60% female, 50% each, 60% male + 40% female) using the software MorphX (http://www.norrkross.com/software/morphx/morphx.php). In total, each of those gender-neutral faces was presented five times to the participants.

Other tasks with faces and words were additionally presented during the experimental sessions; the results are discussed elsewhere (Moellers, 2015). As dependent variables, gender-neutral personality attributes and faces were rated using 100-point VAS ranging from the endpoint *masculin*e (0), to neutral, (50) to the endpoint *feminine* (100) during application of the chemosensory samples.

### Statistical Analyses

The software package SPSS Statistics 22 (Armonk, NY, USA: IBM Corp.) was utilized for statistical analyses. One-sample *t*-tests were performed to investigate odor discrimination performance. Differences of discrimination performance between the pairs of chemosensory stimuli (odor pairs: male–female, female–no odor, male–no odor) was assessed with the help of a repeated-measures ANOVA with the within-subjects factor odor pair and the between-subject factor sex of the rater.

Normal distribution of the rating data was assessed by one-sample Kolmogorov–Smirnov-tests (all *p* > 0.123). Repeated-measures ANOVAs with the sex of the rater (male or female) as a between-subject factor and the sex of the donor (chemosensory samples: male chemosignals, female chemosignals, no odor sample) as a within-subject factor were utilized to analyze chemosensory communication of gender information in odor perception and social perception.

Bivariate Pearson correlations were used to assess associations between masculinity-femininity ratings and hedonic ratings (intensity and pleasantness) of the chemosensory samples. Violations of sphericity were adjusted via Greenhouse–Geisser correction and effect sizes were calculated for *F*-tests ($\eta_{p}^{2}$, partial Eta^2^). Significant main effects and/or interactions were analyzed further using paired-comparison (*t*-tests for two samples and repeated samples) and corrected for multiple comparison using the Bonferroni method. *P*-values < 0.050 were considered significant.

## Results

### Donation Exercise Intensity Analysis

In order to investigate general physical fitness, exercise intensity and associated sex differences, systolic and diastolic blood pressure as well as pulse were analyzed. Pulse varied significantly across measurements, *F*(2,48) = 46.271, *p* < 0.001, $\eta_{p}^{2}$ = 0.658, but not depending on the sex of the donor, *F*(1,24) = 1.545, *p* = 0.226, $\eta_{p}^{2}$ = 0.060. Systolic blood pressure varied significantly across measurements, *F*(2,48) = 14.058, *p* < 0.001, $\eta_{p}^{2}$ = 0.369, and depending on the sex of the donor, *F*(1,24) = 5.352, *p* = 0.030, $\eta_{p}^{2}$ = 0.182. Diastolic blood pressure varied significantly across measurements, *F*(2,48) = 45.628, *p* < 0.001, $\eta_{p}^{2}$ = 0.655, but not depending on the sex of the donor, *F*(1,24) = 1.611, *p* = 0.217, $\eta_{p}^{2}$ = 0.063. A significant interaction was found, *F*(2,48) = 65.728, *p* = 0.037, $\eta_{p}^{2}$ = 0.128.

No sex differences were found for pulse measures. As blood pressure is generally higher in normotensive men compared to women (for a review: Lopez-Ruiz et al., 2008), sex differences in systolic and diastolic blood pressure were found prior to donation (**Table 1**). Physical fitness and exercise intensity after donation were comparable across sexes. In order to classify the strength of the physical activity during the donation, the heart rate (pulse in beats per minute; BPM) after the first donation session, male donors: *M* = 104.21, *SD* = 19.42; female donors: *M* = 110.44, *SD* = 15.43, and after the second donation session, male donors: *M* = 107.52, *SD* = 19.84; female donors: *M* = 116.72, *SD* = 16.79, can be classified as moderate and aerobic exercise zones (Fox et al., 1971).

**Table 1:** Sex-differences in physiological parameters pulse (in BPM) and blood pressure (in mm Hg) for male and female donors.

Physiological parameter		Sex of the donor		Sex difference
		Male	Female	
Pulse	Pre	86.74 (17.07)	94.67 (12.34)	0.135
	Post	107.52 (19.85)	116.72 (16.79)	0.219
Systolic blood pressure	Pre	121.40 (6.94)	112.56 (8.90)	0.008^∗^
	Post	111.40 (8.77)	106.67 (6.24)	0.132
Diastolic blood pressure	Pre	80.69 (5.70)	74.21 (8.81)	0.031^∗^
	Post	66.19 (6.93)	65.64 (4.69)	0.812

*Pre indicates measurements before physical activity, post indicates blood pressure after both 20 min donation sessions. Pairwise comparisons of sex differences of the donors (independent sample *t*-test) with Bonferroni-corrected *p*-values. ^∗^marks significant values *p* < 0.050.*

### Masculinity-Femininity Rating of the Chemosensory Samples

**FIGURE 2:**
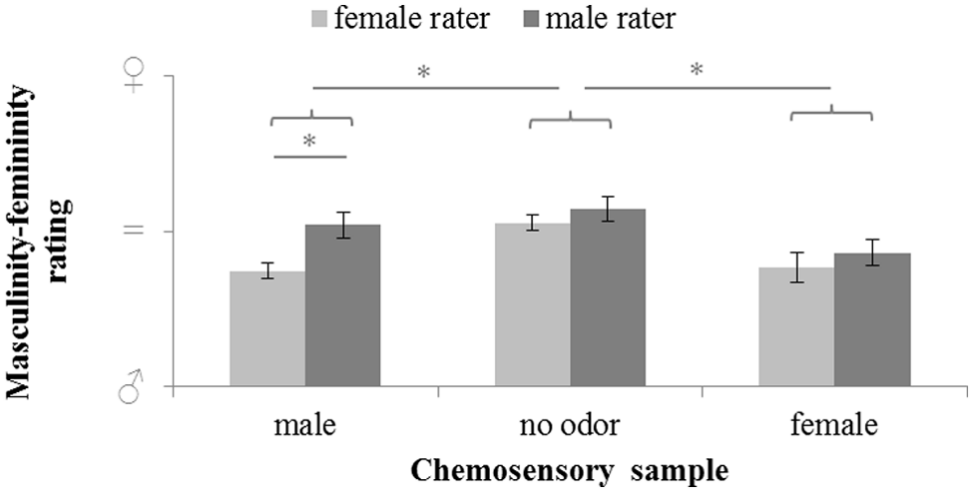
**Masculinity-femininity rating of chemosensory samples via a 100-point VAS by female and male raters (masculine 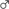 = 0, neutral = 50; feminine 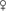 = 100).** ^∗^ marks *p* = 0.001.

Masculinity-femininity rating of body odors varied significantly with sex of the donor (chemosensory samples: male chemosignals, female chemosignals or no odor sample), *F*(2,58) = 9.526, *p* < 0.001, $\eta_{p}^{2}$ = 0.247, and the sex of the rater (male or female), *F*(1,29) = 9.866, *p* = 0.004, $\eta_{p}^{2}$ = 0.254. Overall, exploratory comparisons revealed more feminine ratings of the no-odor samples, *M* = 54.81, *SD* = 10.78, than both female, *M* = 40.77, *SD* = 15.6); *t*(30) = 3.707, *p* < 0.001, and male chemosignals, *M* = 44.42, *SD* = 13.42; *t*(30) = 3.709, *p* < 0.001. Female raters accurately rated male chemosignals as more masculine than male raters, *t*(29) = 3.599, *p* = 0.001; whereas no sex differences were found for the rating of female chemosignals and no odor samples, all *p* > 0.258; **Figure 2**, **Table 2**.

**Table 2:** Mean values and standard deviations, M (SD), for the odor perception task (masculinity-femininity rating) and social perception tasks (gender-neutral personality attributes and faces) by sex of the rater (male and female) and chemosensory sample (no odor, male chemosignals and female chemosignals).

	Sex of the rater					
Chemosensory sample	Masculinity-femininity rating		Personality attributes rating		Face rating	
	Male	Female	Male	Female	Male	Female
Male chemosignals	52.00 (13.17)	37.31 (9.35)	52.39 (3.65)	51.75 (3.05)	55.46 (5.33)	47.05 (7.46)
Female chemosignals	43.17 (10.75)	38.53 (19.18)	50.85 (4.28)	52.64 (4.81)	55.17 (7.35)	46.45 (8.23)
No odor sample	57.10 (11.38)	52.66 (10.07)	53.58 (3.98)	53.40 (3.63)	56.60 (3.54)	42.01 (6.87)

### Hedonic Ratings of the Chemosensory Samples

**FIGURE 3:**
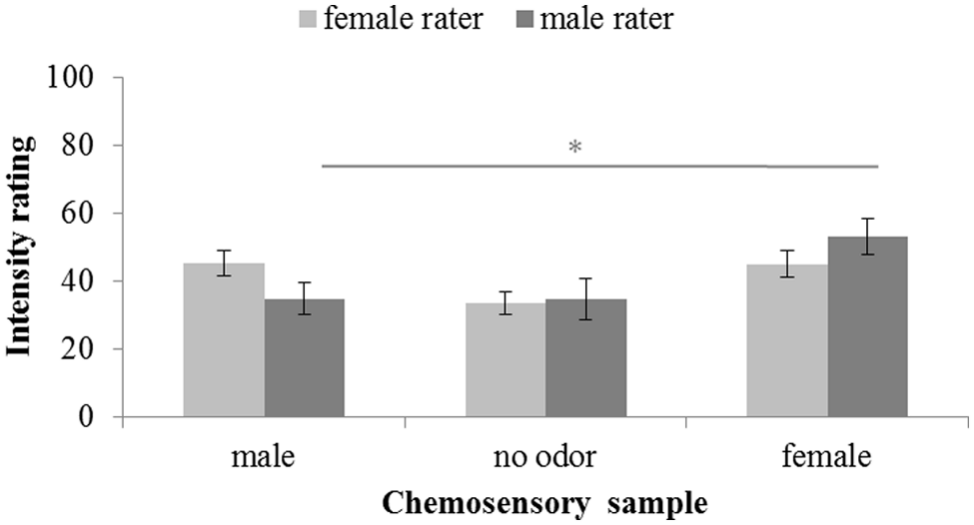
**Intensity rating of chemosensory samples via a 100-point VAS by female and male raters.** ^∗^ marks *p* < 0.010.

Intensity ratings were significantly different across chemosensory conditions, *F*(2,58) = 11.580, *p* < 0.001, $\eta_{p}^{2}$ = 0.238. No main effect of the sex of the rater can be reported, *F*(1,29) = 0.003, *p* < 0.958, $\eta_{p}^{2}$ = 0.000, but a significant interaction between sex of rater and donor was found, *F*(2,58) = 4.059, *p* = 0.022, $\eta_{p}^{2}$ = 0.123. Overall, paired-comparisons revealed no intensity differences between both the male, *M* = 40.26; *SD* = 17.32, and female, *M* = 49.05; *SD* = 18.38, chemosignals, *t*(30) = 2.384, *p* = 0.072, and the male chemosignals and the no odor sample, *M* = 34.19; *SD* = 18.54; *t*(30) = 1.628, *p* = 0.342. Only female chemosignals were perceived to be more intense than the no odor sample, *t*(30) = 5.177, *p* < 0.001. While female raters perceived male chemosignals, *M* = 45.25; *SD* = 15.01, to be as intense as female chemosignals, *M* = 45.06; *SD* = 15.84, *t*(15) = 0.052, *p* = 0.959, male raters perceived male chemosignals, *M* = 34.93; *SD* = 18.49, to be less intense than female chemosignals, *M* = 53.30; *SD* = 20.43; *t*(14) = 3.218, *p* = 0.006, **Figure 3**.

Pleasantness ratings significantly differed depending on the sex of the donor, *F*(1,47) = 11.580, *p* < 0.001, $\eta_{p}^{2}$ = 0.285, but not depending on the sex of the rater, *F*(1,29) = 0.148, *p* = 0.704, $\eta_{p}^{2}$ = 0.005. Exploratory paired-comparisons revealed that female chemosignals, *M* = 38.48; *SD* = 17.04, were perceived to be significantly less pleasant compared to male chemosignals, *M* = 50.48, *SD* = 13.08; *t*(30) = 3.492, *p* = 0.006, and compared to the no odor sample, *M* = 52.05, *SD* = 8.89; *t*(30) = 3.945, *p* > 0.001. Higher pleasantness of female chemosignals is associated with higher femininity, *r* = 0.424; *p* = 0.018, and lower intensity ratings, *r* = -0.599, *p* < 0.001. Odor ratings of male chemosignals were not significantly correlated, all *p* > 0.438. Familiarity ratings did not vary with the sex of the donor, *F*(2,58) = 0.210, *p* = 0.811, $\eta_{p}^{2}$ = 0.007, or the sex of the rater, *F*(1,29) = 0.338, *p* = 0.565, $\eta_{p}^{2}$ = 0.012.

### Discrimination of Chemosensory Samples

During the discrimination task of the odors (twelve trials), the chance level of correct discrimination (33%) equals four correct out of twelve total trials (collapsed across odor conditions) and 1.33 correct out of four trials per odor condition. Participants correctly identified the target odor in 55.89% of all trials, *M* = 6.71, *SD* = 1.95; their general discrimination ability was significantly higher than chance level, one-sample *t*-test: *t*(30) = 7.840, *p* < 0.001. With regards to the different chemosensory conditions, participants were able to discriminate a number of pairs significantly higher than chance level in each odor condition, male-neutral: 58%, *M* = 2.32, *SD* = 0.94; male–female: 47.5%, *M* = 1.90, *SD* = 1.19; female-neutral: 63.75%, *M* = 2.55, *SD* = 0.99; all *t*(30) ≥ 2.68, all *p* ≤ 0.012. Discrimination capacity between the three different odor sample pairs did vary significantly, *F*(2,58) = 3.32, *p* = 0.043. Discrimination between the no odor sample and female chemosignals was significantly better than discrimination between male and female chemosignals, *p* = 0.048. No discrimination differences were found regarding a possible effect of the sex of the rater, *F*(1,29) = 0.063, *p* = 0.804.

### Social Perception

The influence of male and female chemosignals on the perception of gender-neutral personality attributes revealed a main effect of the sex of the donors, *F*(2,58) = 3.967, *p* = 0.024, $\eta_{p}^{2}$ = 0.120, but not of the raters, *F*(1,29) = 0.073, *p* = 0.789, $\eta_{p}^{2}$ = 0.003. No difference was found between male and female chemosignals, *t*(30) = 0.385, *p* = 0.703. However, compared to the neutral sample, male and female chemosignals were both associated with more feminine adjective ratings, male: *t*(30) = 2.716, *p* = 0.011; female: *t*(30) = 2.383, *p* = 0.011, **Table 2**.

**FIGURE 4:**
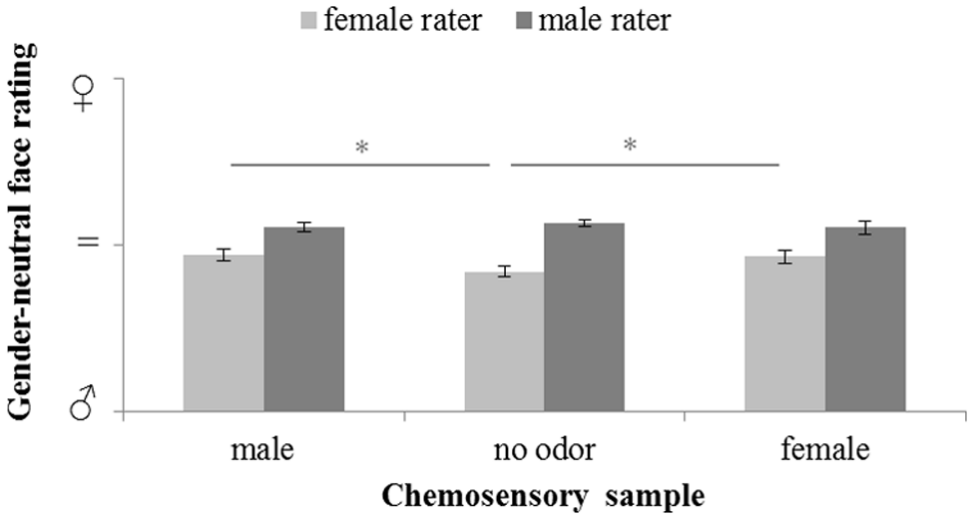
**Masculinity-femininity rating of gender-neutral faces via a 100-point VAS during exposure to chemosensory samples by female and male raters (masculine 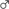 = 0, neutral = 50; feminine 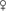 = 100).** ^∗^marks *p* < 0.050.

The influence of male and female chemosignals on the perception of gender-neutral faces (**Figure 4**) did not reveal a main effect of the sex of the donors, *F*(2,58) = 1.685, *p* = 0.194, $\eta_{p}^{2}$ = 0.055. However, a significant main effect of the sex of the raters, *F*(1,29) = 27.152, *p* < 0.001, $\eta_{p}^{2}$ = 0.484, and a significant interaction of the sex of the donors and raters, *F*(2,58) = 4.892, *p* = 0.011, $\eta_{p}^{2}$ = 0.144, was yielded. Paired-comparison revealed that male raters generally rated the faces as more feminine than female raters, all *p* ≤ 0.004. Faces were perceived equally gender-neutral under exposure of female and male chemosignals, *t*(15) = 0.402, *p* = 0.691. While, for male raters, there were no rating differences across chemosensory conditions, all *p* ≥ 0.412, female raters evaluated gender-neutral faces as significantly more feminine (**Table 2**) when exposed to male, *t*(15) = 3.359, *p* = 0.004, and female chemosignals, *t*(15) = 3.010, *p* = 0.009, compared to the no odor sample. Ratings of male and female chemosignals did not differ, *t*(15) = 0.328, *p* = 0.747.

### Affective Introspection

For the PANAS subscales, no main effects of the sex of the donor or rater were found. Only sample- and sex-unspecific stabilizations of the raters’ mood were found after the experimental procedure, both for positive mood, *F*(1,28) = 15.220, *p* = 0.001, $\eta_{p}^{2}$ = 0.352; pre: *M* = 53.3, *SD* = 13.65; post: *M* = 48.57, *SD* = 15.91, and for negative mood, *F*(1,28) = 4.220, *p* = 0.049, $\eta_{p}^{2}$ = 0.131, pre: *M* = 10.89, *SD* = 8.13; post: *M* = 9.63, *SD* = 8.69.

### Chemosensory Induced Judgment Bias

The judgment bias induced by male and female chemosensory samples on odor perception and social perception is further investigated. *Post hoc*, judgment bias was calculated by subtracting the rating of the no odor sample from the rating of the chemosensory sample (male or female chemosignal). Using this method, a masculinity bias was represented by a negative value and a femininity bias was represented by a positive value. A value not different from 0 represents no bias.

**FIGURE 5:**
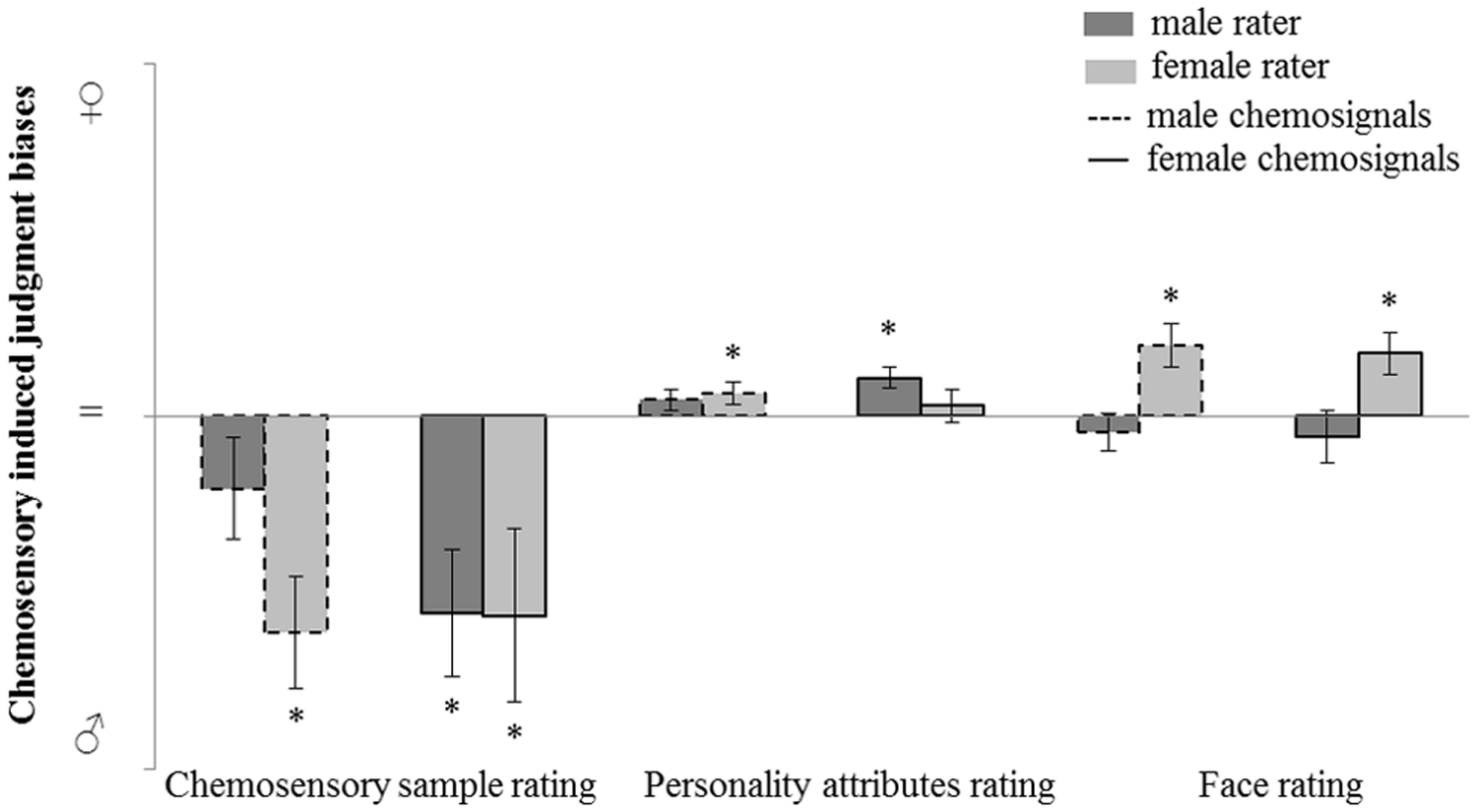
**Judgment bias in male and female raters induced by male and female chemosensory samples for odor perception (masculinity-femininity rating) and social perception (gender-neutral personality attributes and faces rating tasks).** No odor-baseline controlled sample rating charted on a bias scale: masculinity bias (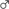; chemosensory sample rating > no odor rating), no bias (=; chemosensory sample rating = no odor rating), femininity bias (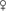; chemosensory sample rating < no odor rating). ^∗^marks *p* < 0.050.

For the main odor perception and social perception tasks (i.e., masculinity-femininity rating of the chemosensory samples, gender-neutral personality attributes and faces rating tasks), judgment bias variables were calculated and chemosensory induced judgment bias was assessed via one sample *t*-tests (test value = 0; **Figure 5**).

For the odor perception task, a masculinity bias induced by male chemosignals was found in female raters, *M* = -15.34, *SD* = 15.77; *t*(15) = -3.893, *p* = 0.001, but not in male raters, *M* = -5.10, *SD* = 14.03; *t*(14) = -1.408, *p* = 0.181. A masculinity bias induced by female chemosignals was found in female raters, *M* = -14.13, *SD* = 24.67; *t*(15) = -2.290, *p* = 0.037, and in male raters, *M* = -13.93, *SD* = 17.32; *t*(14) = -3.116, *p* = 0.008.

The same analysis was computed for the two social perception tasks. For the rating of gender-neutral personality attributes, a femininity bias induced by female chemosignals was found in male raters, *M* = 2.23, *SD* = 2.85; *t*(14) = 3.709, *p* = 0.002, but not in female raters, *M* = 0.75, *SD* = 4.72; *t*(15) = 0.637, *p* = 0.533. A femininity bias induced by male chemosignals was found in female raters, *M* = 1.64, *SD* = 3.01; *t*(15) = 2.182, *p* = 0.045, but not in male raters, *M* = 1.19, *SD* = 2.90; *t*(14) = 1.589, *p* = 0.134.

For the rating of gender-neutral faces, a femininity bias induced by female chemosignals was found in female raters, *M* = 4.44, *SD* = 5.91; *t*(15) = 3.010, *p* = 0.009, but not in male raters, *M* = -1.43, *SD* = 7.26; *t*(14) = -0.763, *p* = 0.548. A femininity bias induced by male chemosignals was found in female raters, *M* = 5.04, *SD* = 6.00; *t*(15) = 3.359, *p* = 0.004, but not in male raters, *M* = -1.14, *SD* = 5.22; *t*(14) = -0.846, *p* = 0.412.

## Discussion

The present study aimed at establishing how humans communicate sex-specific information via body odors involving heterosexual male and female donors and raters. Healthy normosmic males and females were exposed to male and female chemosignals and no odor samples. First, a masculinity bias in human body odor perception was found in a masculinity-femininity rating, hedonic ratings and sensory-based discrimination of the chemosensory samples. Second, human body odor modulated the perception of gender-neutral faces and personality attributes toward a femininity bias.

Concerning the chemosensory communication of sex-specific information via male and female chemosignals in male and female raters, we found that female chemosignals are judged as rather intense and unpleasant by male raters. Although men and women are able to perceive sensory-based differences, the sex of the donor cannot be established from such stimuli. Both men and women seem to judge any body odor as rather masculine. Based on exploratory analyses, female raters are more accurate than male raters in assigning male body odor to a male donor, suggesting that mainly females detect the masculinity in male body odors.

Our result of a negative correlation between intensity and pleasantness of female body odor (i.e., the more intense, the more unpleasant the perception of female body odor) is in partial accordance with findings of Doty et al. (1978) where inversed pleasantness and intensity ratings were found for female and male axillary odor. This pattern is not restricted to body odor and was also reported cross-culturally for food and everyday odorants (Distel et al., 1999). Besides the inability to establish masculinity or femininity features based on body odor alone, we conclude that the scent of human body odor seems to be closely associated to the male gender. Two reasons might explain why body odor is perceived as rather masculine. First, perceivable and intense body odor as a consequence of physical activity and strength might cue masculine gender stereotypes of dominance and power. A masculinity bias in sex identification might rely on a semantic tendency of strength being related to masculinity (Doty et al., 1978) – a tendency that might specifically affect female raters presenting higher olfactory abilities. Second, exposure to female body odor might be less frequent than exposure to male body odor. As females purchase and apply fragrances more often and perceive artificial fragrances as more arousing than males (Herz and Cahill, 1997), a diminished exposure to female body odor for both males and females might be the result. Along these lines, a decreased number of opportunities where determining body odor as originating from male or female donors might go along with an inhibited learning process of differentiating female and male body odor. Assuming that – in a social context – the source of a body odor is not clearly identifiable and the scent is less likely to have a female sender, the most adapted response would be to identify the scent as masculine. Another reason for diminished exposure to female body odor might be the decreased intensity compared to male body odor in relation to biological factors. Male body odor is often perceived as more intense and less pleasant than female body odor (Doty et al., 1978; Mitro et al., 2012), an effect that can be related to stronger axillary secretion (Sergeant, 2010) and a higher concentration of sweat-degrading skin bacteria (Jackman and Noble, 1983), steroid hormones, or axillary hair in men. In light of the previous studies and in accordance with our results, we conclude that an unequivocal sex identification based on body odor alone is unlikely to be performed by individuals from industrialized societies.

Investigating the possible modulating effects of chemosensory samples in male and female raters on social perception and self-perception, we aimed to clarify whether sex-specific information in body odors modulate the evaluation of ambiguous conspecifics (personality attributes and faces) rather than modulating affective introspection (mood rating). Supporting the idea that body odors – as social signals – affect the perception of conspecifics rather than introspection, we found that exposure to any body odor induced a femininity bias in social perception. No sex-specific chemosensory effect on a rater’s mood was detected. When exposed to any body odor, men and women rated gender-neutral personality attributes as more feminine. Additionally, we found that women perceived gender-neutral faces to be more feminine. Body odors seem to facilitate social cognition and induce a femininity bias, which might be explained by the idea that chemosignals are representatives for social and emotional situations. Evidence arises from neuroimaging studies during which females exposed to chemosignals activate brain areas involved in the assessment of a human quality of chemosensory cues (Zhou and Chen, 2008). Furthermore, conspecifics’ body odor is processed in the amygdala and insular regions, unlike other non-human odors (Lundström and Olsson, 2010). Additionally, a strong neural connection of chemosensory processing areas and emotionally relevant limbic areas exists (Albrecht and Wiesmann, 2006). These findings suggest that successful chemosensory communication with a conspecific requires an accurate assessment of emotional cues. Along this train of thought, the femininity bias might be a result activated by emotional sensitivity that is stereotypically associated with feminine referents. As females are more receptive to subtle emotional signals (Pause et al., 2004; Radulescu and Mujica-Parodi, 2013), we find here that the dominant visual signal is modulated more by the chemosensory signal than in male raters.

Taken together, we suspect task-related differences might have led to the sharp contrast of masculinity and femininity biases found. The masculinity bias was established in sex identification via masculinity-femininity ratings and evaluation of the chemosensory samples. The femininity bias was induced by the chemosignals in social perception tasks. Here, two different perceptive and cognitive processes (olfactory perception and evaluation versus multisensory integration and higher-order evaluation) are involved and might explain the opposing findings. While the masculinity bias becomes evident during evaluation of chemosensory information, the femininity bias appears when the olfactory information is a modulating source of information while performing a masculinity-femininity rating on ambiguous visual stimuli. We therefore assume that, besides sex and gender, task complexity might have played an important role on the gender-related biases in chemosensory information transmission.

Addressing the limitations of the present study, we acknowledge methodical limitations in relation to the donation method. Taking into account that different axillary glands are contributing to odorous secretions, we are aware that mainly thermoregulative eccrine glands were stimulated. However, based on knowledge of apocrine and eccrine hyperhidrosis, eccrine gland activity is involved in the transportation of odorous (sebaceous and apocrine) sweat (Hurley, 1989). Also, acknowledging the presence of apoeccrine glands (that are as high in number as apocrine and eccrine glands; Labows et al., 1999), we believe that a stimulation of apocrine and eccrine glands during the experimental set-up resulted in a complex mixture of chemical compounds. Chemosignals were grouped in donor pools characterized by one consistent characteristic: the sex of the donor. The benefit of the superdonor pool in homogenizing across entire group samples, however, represents the inconvenience of being unable to track the individual donor’s quadrants that contributed to the chemosensory sample of each receiver. The donation method involving short periods of physical activity was chosen over continuous body odor collection throughout the day for two reasons. First, the entire donation in a laboratory setting assures that no uncontrolled psychological or emotional factors bias the chemosensory samples. Second, to assure that circadian hormonal variations between sexes do not influence the quality of the chemosensory samples (Mong et al., 2011), donation appointment times were kept short and constant for all donors. Additionally, while we controlled for the presence of axillary hair, we are not able to rule out that chemosensory samples might have differed in association with the length of axillary hair (Kohoutová et al., 2012). We find an inverse intensity rating, and we think that the results might be influenced by axillary hair length.

We acknowledge that influences of hormonal contraception were controlled in female donors and raters and influences of menstrual cycle were kept constant in female raters, although not systematically studied. Menstrual cycle differences for female donors were controlled for by using pooled donor sets and, for every female rater, all testing sessions were placed in the same menstrual cycle phase. Female raters in fertile menstrual cycle phases show preferences for symmetrical (Gangestad and Thornhill, 1998) and dominant (Havlicek et al., 2005) body odor of male donors and exhibit an increased chemosensory sensitivity (Doty, 1981). Additionally, only pleasantness and preference ratings were affected by menstrual cycle, while across all cycle phases female raters did not report intensity differences in male body odor (Rantala et al., 2006). Male raters show a preference for ovulatory female body odor (Singh and Bronstad, 2001) only when female donors do not take hormonal contraception (Kuukasjärvi et al., 2004).

Formulating a distinct suggestion on the inclusion of male and female body odor donors in future chemosensory research, we would like to emphasize the importance of the inclusion of female chemosignals when performing chemosensory research on emotional, social, or sexual behavior in humans. To date, male chemosignals are most widely studied in emotional chemosensory communication research, with the argument that their body odor is not affected by menstrual cycle phases. While emotional introspection in a rater does not seem to be affected by the sex of a donor, emotional communication in female raters might still be biased. Observing a masculinity bias in body odor perception and a femininity bias introduced by chemosignals during social perception, we would like to encourage further research to disentangle the influence of cyclic fertility on chemosensory communication of sex and gender information in social perception.

## Author Contributions

All authors made substantial contributions to the conception or design of the work. EM performed data acquisition. Data analysis and interpretation was performed by SM, EM, and JF. Drafting of the manuscript was performed by SM. Revising and approving the final version of the manuscript was accomplished by all authors.

## Conflict of Interest Statement

The authors declare that the research was conducted in the absence of any commercial or financial relationships that could be construed as a potential conflict of interest. The reviewer, Katrin Lübke, and handling Editor declared their shared affiliation, and the handling Editor states that the process nevertheless met the standards of a fair and objective review.
